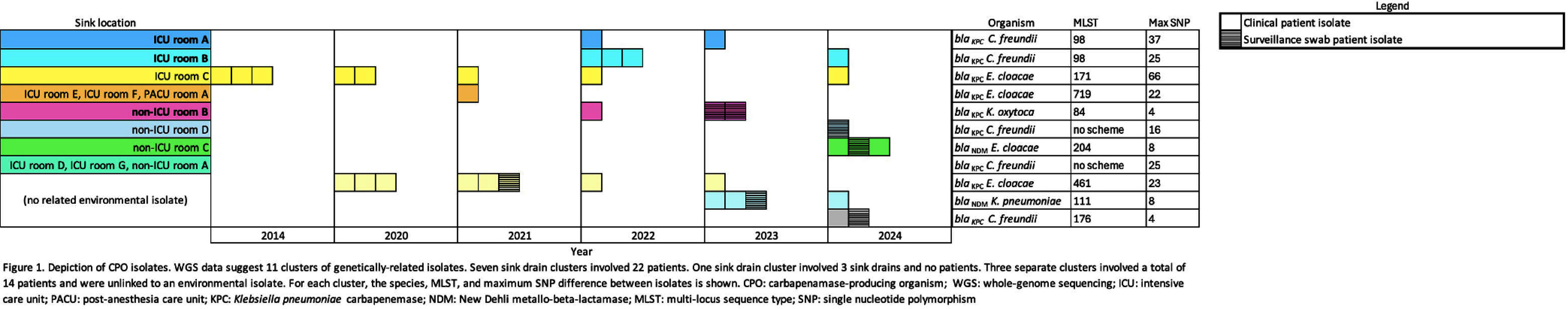# Impact of CPO Premise Plumbing Contamination on CPO Transmission within a Minnesota Acute Care Hospital

**DOI:** 10.1017/ash.2025.357

**Published:** 2025-09-24

**Authors:** Laura Tourdot, Christine Lees, Bradley Craft, John Kaiyalethe, Kari Gand, Megan Krieglmeier, Jesse Sutherland, Terra Menier, Jessica Kanelfitz, Mindy McFarren, Ginette Dobbins, Susan Kline, Patricia Ferrieri, Paula Snippes Vagnone, Jennifer Dale, Krista Knowles, Tara Suhs, Alison Galdys

**Affiliations:** 1 Minnesota Department of Health; 2 Fairview Health Services; 3 M Health Fairview; 4 Minnesota Department of Health; 5 University of Minnesota; 6 MN Dept. of Health, Public Health Lab; 7 MDH; 8 University of Minnesota Medical Center

## Abstract

**Background:** Carbapenem-resistant Enterobacterales (CRE) are reportable statewide with required isolate submission to the Minnesota Department of Health (MDH) Public Health Laboratory (PHL), where carbapenemase production and mechanism identification is confirmed. MDH reviews all detected carbapenemase-producing organisms (CPOs) for potential transmission. Suspected transmission clusters are assessed for relatedness using whole genome sequencing (WGS). In 2022, increased detection of multiple bacterial genera of Klebsiella pneumoniae carbapenemase (KPC)-CRE occurred at acute care hospital-A, (ACH-A) and in 2023 the increase in KPC-CRE was accompanied by an increase in New Delhi metallo-β-lactamase (NDM)-CRE detection. **Methods:** MDH partnered with ACH-A to review increased CPO detection. MDH-PHL conducted WGS including multilocus sequence typing (MLST) and single nucleotide polymorphism (SNP) analysis on isolates. WGS suggested clusters of relatedness spanning multiple years and epidemiologic data revealed common room occupancy. Infection prevention and control (IPC) principles were reinforced in cluster areas and audits verified adherence, prompting consideration of an environmental reservoir. An environmental screening plan was developed focusing on sink drains from common rooms. In May 2024, 94 swabs from sink drains were collected and CPO culture-based screening was conducted using selective media followed by molecular testing of bacterial growth by MDH-PHL. **Results:** There was detection of CPOs from 28 of 94 (29.8%) sink drains. Eight environmental KPC-CRE isolates and one NDM-CRE isolate appeared genetically related to 22 unique patients over a 10-year period (Figure 1). Three sink drain isolates showed genetic similarity to each other, but not to patient isolates. Three CPO clusters, representing 14 patients, had genetically similar isolates without an associated environmental isolate. However, isolates were collected over months to years suggesting an undetected reservoir. In August 2024, ACH-A initiated mitigation strategies to prevent CPO transmission from environmental reservoirs, including modification of sink plumbing, maintaining a splash zone, refraining from disposal of bodily fluids in sinks, optimizing sink hygiene, and monthly screening of inpatients in units with known CPO sink contamination. From August to December 2024, 325 patients were screened with 1.2% of specimens detecting KPC-CRE colonization. **Conclusion:** Sink drains containing CPOs on multiple hospital units that correlated with patient cases were identified at ACH-A. WGS suggests intermittent transmission of different CPOs over 10 years, and clusters of transmission appear to be related to environmental sources. Strict implementation and adherence to IPC measures, including those that minimize the spread of CPOs from facility premise plumbing, are critical to prevent CPO transmission despite widespread premise plumbing contamination.

**Figure:**